# The Impact of COVID-19 and Homeschooling on Students' Engagement With Physical Activity

**DOI:** 10.3389/fspor.2020.589227

**Published:** 2021-01-26

**Authors:** Astrid Roe, Marte Blikstad-Balas, Cecilie Pedersen Dalland

**Affiliations:** ^1^Department of Teacher Education and School Research, University of Oslo, Oslo, Norway; ^2^Department of Primary and Secondary Teacher Education, Oslo Metropolitan University, Oslo, Norway

**Keywords:** education, home-schooling, remote teaching, COVID-19, physical activity (exercise)

## Abstract

The COVID-19 pandemic forced an unprecedented global shutdown that closed schools for months. In many nations, schools were closed to students, and teachers directed educational activities remotely via digital devices or homeschooling resources. This article explores how these months of homeschooling have affected the physical activity of Norwegian students in Grades 1–10. All Norwegian students are supposed to have at least 60 min of physical activity every day in school. We draw on data from two surveys, one with parents (*N* = 4,624) and the other with teachers (*N* = 726), to provide an indication of the daily physical activity students engaged in during the period of homeschooling. An important finding from the teacher survey is that the degree to which schools prioritized physical education among the school subjects varied greatly between schools and teachers. Key findings from the parent survey were that reported time spent on physical activity increased with the age of the students, that many parents expressed concerns about increased sedentary behavior, and that the most active students were those who showed the greatest engagement and effort in schoolwork in general. Our findings raise the questions of whether students were given too much responsibility for their own physical activity during this period and whether teachers should provide their students with more digital workout sessions and instructional videos.

## Students' Physical Activity During Digital Homeschooling

The COVID-19 pandemic has forced an unprecedented global shutdown that has greatly changed what it means to be a teacher, a student, and even a parent in the months that schools have been closed. While most school systems normally require daily physical attendance during week days and bring students together in large groups to learn in a collective endeavor, the closing of schools and the months of social distancing have shifted the site of learning to the home, where learning happens primarily alone or with the help of family members through the technologies available. Physical education (PE) and organized active recess time that would normally take place in school have been either canceled completely or made a part of digital homeschooling. Social distancing measures, in Norway, require people to stay at least 1 meter apart from each other and avoid gatherings with anyone other than their closest family members. These measures have resulted in the cancelation of all organized afterschool sporting activities. Across the world, millions of students have experienced the disruption of their normal routines for learning and physical activity (PA). As Krumsvik (2020) noted, it is important for educational researchers to investigate different aspects of the educational consequences of the COVID-19 crisis, in order to avoid the domination of anecdotal evidence about how the shutdown has impacted students' lives. This is particularly important as the World Health Organization (WHO, 2020) has predicted more global pandemics in the future.

This article sheds light on how COVID-19 and the closure of schools impacted young people's physical activity (PA) during the periods with closed schools and home schooling. Previous research has documented that PA plays an important role in physical and psychosocial health and wellbeing for children and young people (Biddle et al., 2019). Research has shown that a sedentary lifestyle in students is associated with chronic diseases later in life, as well other health-related risk behaviors such has unhealthy dietary patterns (Carson et al., 2016; Tremblay et al., 2016). Globally, there is general concern about the low amount of PA and the increase in sedentary behavior in young people as well as child obesity (UNICEF, 2019). While COVID-19 is a global pandemic leading to a global shutdown, it is also evident that the pandemic has affected different nations, regions, and sociodemographic groups in different ways and with different intensities. Young people's education has been affected in ways, which are as yet not fully appreciated.

In this article, we report on how school shutdown has affected the PA of students in Grades 1–10 across Norway. Teachers throughout the country were asked to perform their teaching from home, through digital devices and remote teaching (Krumsvik, 2020). As in other countries, Norwegian teachers and school leaders were not prepared to go digital overnight, despite good technological infrastructure (Blikstad-Balas and Klette, 2020). Drawing on surveys administered to both parents (*N* = 4,642) and teachers (*N* = 726) about how digital homeschooling was organized, we have investigated what kind of (digital) attendance school has required from students in different grades during the shutdown and the estimated PA they have engaged in during the period of homeschooling. We also mapped what parents and teachers considered to be the main challenges and benefits of homeschooling. In this article, we mainly emphasize challenges and benefits regarding the students' opportunities to stay physically active.

## COVID-19 and Education: Great Variation within Nations and Schools

In April 2020, the United Nations Children's Fund (UNICEF) reported that about 3 billion people were in lockdown around the world and that almost 90% of the student population was cut off from school (Winther and Byrne, 2020). Closing schools has a range of adverse consequences, ranging from disrupted learning, food insecurity for children who rely on open schools for access to healthy food, and increased exposure to violence and exploitation alongside the challenges of creating, maintaining, and improving distance learning (UNESCO, 2020).

Around the world, attempts to continue providing education to students varied greatly, due to significant differences in access to technologies, which could support remote teaching, such as books, TV, smartphones, and the internet. For example, while children in Rwanda received pedagogical content through the use of radio, several countries, including Côte d'Ivoire, arranged TV classrooms, an initiative including taping lessons to be aired on national TV (Miks and McIlwaine, 2020). Many international providers of educational tools made their resources free of charge during the pandemic, and UNICEF launched the #LearningAtHome initiative, which provided activities every day that parents could adapt and share with others, given that they had internet access (Miks and McIlwaine, 2020). Although, little is known about what kind of schooling children actually took part in during the pandemic, it appears that most countries attempted to provide relevant tasks for children to do at home, often in combination with pedagogical content provided through TV, radio, or the internet.

Inadequate digital infrastructure in schools and society are key barriers to the successful implementation of educational information and communications technology (ICT) strategies (Bingimlas, 2009; Gil-Flores et al., 2017). Therefore, Norway is a particularly interesting case when it comes to education and COVID-19, due to the vast technological infrastructure available. Internet access at home in the population has repeatedly been measured at 98% (e.g., Statistics Norway, SSB, 2020a). ICT infrastructure is an obvious prerequisite for integrating digital technology into instruction, something that would greatly facilitate teaching when students are distanced from their classrooms. Since the 1990s the question of access has dominated the discourse around ICT in many countries, and many schools have reported pressure to provide one-to-one (1:1) access for all their students, that is, one digital device per student provided by the school (Blikstad-Balas and Davies, 2017). However, several studies have shown that access is not a reliable predictor of teachers' actual implementation and uptake of digital technology (Gil-Flores et al., 2017; Blikstad-Balas and Klette, 2020). The newest Teaching and Learning International Survey (TALIS) report from Norway highlighted as critical the discrepancy between merely providing access for students and preparing teachers to actually utilize the technology in their everyday teaching (Throndsen et al., 2019). The overall access to technology for students in Norway has been consistently high and significantly above the European average for the student-per-laptop ratio (OECD, 2015).

Both primary and secondary students and teachers in Norway have access to digital technologies (Dalaaker et al., 2012; Egeberg et al., 2012; Fjørtoft et al., 2019). While 1:1 access is the norm in upper secondary schools, lower secondary schools can also either provide permanent 1:1 access or lend students laptops or tablets for use in a specific lesson. In summary, the access to ICT in Norwegian schools enables broad use of ICT in the classroom, and the national curriculum explicitly places this responsibility on all teachers across all grades. However, previous classroom research has revealed that the actual uptake of technology varies and is largely dependent on individual teachers. This backdrop provides important contextualization when investigating the degree to which students and parents report that they prioritized PA during the COVID-19 outbreak.

Norwegian teachers have considerable autonomy in deciding how to teach their subject by determining matters such as what pedagogical methods and resources, such as apps and software, to include (Mølstad and Karseth, 2016; Sivesind and Wahlström, 2016). Thus, the general differences between the teachers' practices may be significant within the same school district and even the same school. For better or worse, each teacher has great freedom in determining the pedagogical choices in their classroom. This freedom extends to the use of ICT for pedagogical purposes. Previous research from Norway has found that, even though access to ICT is high and the curriculum is the same, there are big differences in how digital technology is used between schools and also between teachers within the same school (Hatlevik et al., 2009; Egeberg et al., 2012, 2016).

## PA Among Students in Norway

As in many other countries and in line with international recommendations, the Norwegian education system places expectations on schools regarding PA through the subject of physical education (PE) and by stressing that children should be active during the school day (Schmidt et al., 2020). In fact, in 2017, the Norwegian parliament decided that all school children should have at least 60 minutes of PA during the school day, every day. This decision resonates not only with all the research showing general health benefits from PA, but also with research suggesting that PA can have positive effects on students' behavior in class and even on academic outcomes (Norris et al., 2015, 2018). This expectation for PA during the school day is fairly new, and systematic research is necessary to determine how schools are meeting these new requirements. At present little is known about how the time available for PA is used and what forms of PA schools organize for students. It is therefore not possible to precisely estimate the total amount of PA students engage in at different grade levels across Norway, but in addition to general PA, the PE subject has 478 hours per year in Grades 1–7 (students aged 6–12) and 223 h per year in Grades 8–10 (students aged 13–16).

Despite the lack of detailed knowledge about the general PA level among students in Norway across different grade levels, previous studies have indicated that Norwegian students will engage in PA as a direct consequence of schooling during traditional open schools (not distance learning). There are several reasons for this. Only one of four children aged 6–12 are taken to school by car by their parents daily. Two of three walk or use a bicycle, and the rest take public transportation due to the long distance between school and home (NHI Norsk Helseinformatikk, 2015). Students have recesses and play time during the school day, and they have PE as a subject every week (normally between 1.5 and 2.5 h a week). Additionally, many teachers give their students short PA breaks both in the classroom and outdoors, especially in the lower grades. With the exception of inner-city schools, many Norwegian schools are situated in rural surroundings or near wooden areas and parks, which are often used for outdoor teaching. Furthermore, the vast majority of 6–15-year-old students participate in afterschool organized sports one or more days a week (Hammer, 2017). Therefore, while schooling may contribute to more PA, some research has indicated that traditional classroom lessons are the most sedentary and least active segment of a young person's day (Nettlefold et al., 2011; Bailey et al., 2012). This latter point is particularly interesting for this study, as homeschooling could decrease the school-related PA. But it could also give teachers and families the chance to prioritize PA to a larger extent due to the removal of physical barriers to PA at school, such as the number of students in the same physical classroom and the tight schedule of curriculum activities from many teachers.

Even though Norwegians in general are rather healthy compared with many other countries (National Institute of Public Health, 2018), there are also concerns about the general decline in PA in the Norwegian population. For example, Dalene et al. (2018) conducted a longitudinal study with representative samples of 9- and 15-year-old students and identified a large decline in PA for the participants followed from age 9 to 15 years. Several reports on health and wellbeing among young people have found that PA declines with age (Samdal et al., 2016; Hammer, 2017; Bakken, 2019). Further, they have identified a tendency for students with lower socioeconomic status to quit organized PA after school and to have difficulties staying as active as when participating in football, skiing, bicycling, or other forms of organized afterschool PA (Bakken, 2019).

## Methods

The authors developed two surveys about homeschool for children in primary and lower secondary school during the COVID-19 outbreak, one for parents and one for teachers. Both surveys were anonymous and followed the ethical guidelines for participation regulating research in Norway.

The surveys were distributed to parents and teachers digitally because the timing of the survey was crucial: we wanted parents to respond during the period of homeschooling and school lockdown, not in retrospect. Our inclusion criteria were parents with students in Grades 1–10 and teachers teaching in Grades 1–10. There is no systematic way to assemble a traditional random probability sample of people who fit the criteria through the internet, and all internet surveys thus rely on contacting relevant respondents, for example by email or social media, and asking them to complete the survey. As with many other one-time internet surveys, we had to opt for a non-probability convenience sample (Fowler, 2009; Patton, 2015) where we invited participation from whoever saw the survey invitation online. We recruited teachers and parents either through their school owners, who sent out emails to all teachers in their school area offering the possibility to participate, or through social media groups for teachers and parents on Facebook or Twitter. As with any non-probability-based sample, the greatest limitation is the unknown relationship between the sample and the population and the missing theoretical basis for estimating the repetitiveness of the sample. We have included several background variables about the respondents (e.g., where they live and their educational background) to systematically monitor these variables in our samples and compare them with nationally representative samples.

The teacher survey was answered by 726 teachers from most parts of the country. The teacher survey did not include items directly addressing the students' PA or the teaching of PE during lockdown, except for one question about whether any subjects received less attention than usual during the period of homeschooling. Therefore, we primarily draw on data from the parent survey and only report the findings of teachers' responses on the one item concerning school subjects.

The parent survey was answered by 4,642 parents from all over the country. A total of 262 of the country's 365 municipalities were represented with good geographical distribution, and the respondents represented different categories of large and small towns, municipalities, villages, and rural areas. If the parents had several children in primary or lower secondary school, they chose one of their children prior to starting the survey and answered all questions for that child.

The main ambition of the surveys was to investigate all aspects of homeschooling, including what kind of remote teaching students were offered and how parents and teachers experienced the homeschooling situation. The parent survey had background questions about the school location, the student's gender and grade, and the parent's level of education and work situation during the period (work outside home, home office, laid off/unemployed, and stay-at-home parent). After completing the background information, parents answered 24 questions related directly to the homeschool situation, such as digital equipment, attendance requirements, communication with teachers, tasks, subjects, students' engagement and efforts toward schoolwork, and the parent's own experience during the period of homeschooling.

One question in the parent survey directly addressed PA and was used to investigate possible differences in physical activities between groups of students. The question was “How much time does the student spend being physically active during a typical homeschool day, in addition to any exercises in PE?” The response alternatives were “No PA,” “15 min or less,” “15–30 min,” “30–60 min,” and “More than 60 min.” Two open-ended questions asked about concrete positive and challenging experiences during this period. These questions were not aiming at particular aspects (e.g., concerning PA), but 65 parents mentioned PA under the positive rubric, and 109 parents mentioned PA under the challenging rubric. We will provide examples from these responses in the result section.

In this article, we have investigated possible differences between groups based on grade, gender, parents' education and school location in terms of students' voluntary PA. One composite variable describing students' engagement and effort in schoolwork was also analyzed in relation to students' voluntary PA.

For the background variables grade, gender, parents' education, and school location, we used descriptive statistics to calculate the percentage of distribution of time spent on PA among groups within each variable. Practically meaningful differences between groups were chi-square tested to determine statistical significance (*p* < 0.01).

The composite variable measuring engagement and effort was constructed by means of eight items describing students' engagement and effort—or lack of such—regarding schoolwork in this period. For each item, the parents reported on the following five-point Likert scale ranging from “Never” to “Always” to describe how frequent what was described in each item occurred. In order to measure mean values related to PA, the scale was treated as a quasi-interval scale with values from 1 (*never*) to 5 (*always*). Four items described positive engagement and effort, and four items described lack of engagement and effort.

The items regarding positive engagement and effort were the following:

*The student works well with tasks at home*.
*The student has immersed him/herself in schoolwork*.
*The student has liked/enjoyed schoolwork better than usual*.
*The student has been able to read and write more based on interests*.

The items regarding lack of engagement and effort were the following:

*The student skips schoolwork*.
*The student postpones schoolwork until the parents push him/her to do it*.
*The student is struggling to get started with schoolwork*.
*The student finds it difficult to do the schoolwork without help*.

We created a composite score for these eight items as an expression of students' engagement and effort in schoolwork. First, we reverse coded the four items describing lack of engagement and effort. Second, Cronbach's alpha (Cronbach, 1951) was used to measure internal consistency, or how closely related the eight items within the concept were as a group. Cronbach's alpha (α = 0.86) indicated that the combination of items had acceptable internal consistency (George and Mallery, 2016). The mean value for the concept was standardized to 0 and a standard deviation of 1. 95% confidence interval is marked with 1.96 × SE (standard error) in each direction from the mean (**Figure 4**). ANOVAs were used to test whether students' engagement and effort varied across levels of PA.

We coded the open-ended questions in the surveys qualitatively using conventional content analyses (Hsieh and Shannon, 2005), where patterns in responses were identified and aggregated before examples illustrating typical responses were selected. Two of the authors coded all the open-ended questions together and checked for consistency in the coding during this process. Further, any borderline cases or responses that were difficult to code were discussed before deciding on final coding.

## Results

### Background Variables

Table 1 shows the number of students on each grade level in the parent survey. A total of 52% of the respondents represented students at the primary school level (Grades 1–4), 30% students at the intermediate level (Grades 5–7), and 18% students at the lower secondary level (Grades 8–10). Additionally, 96% of respondents had children in public schools and 4% in private schools. The gender distribution showed that parents' responses were about more boys (54%) than girls (46%). In the following analyses, we have divided the students into the following three groups: Grades 1–4, Grades 5–7, and Grades 8–10.

**Table 1:** Distribution of students on each grade level.

	**1st grade**	**2nd grade**	**3rd grade**	**4th grade**	**5th grade**	**6th grade**	**7th grade**	**8th grade**	**9th grade**	**10th grade**	**Total**
*N*	566	589	667	580	568	455	370	348	251	248	4,642
%	12.19	12.69	14.37	12.49	12.24	9.80	7.97	7.50	5.41	5.34	100.0

As a measure of socioeconomic status, we asked parents about their highest level of education (Table 2). Compared to the national average for parents between 25 and 50 years, which we assume represents most of the parents in our sample, our sample has a higher percentage of parents with a master's degree or a PhD and a lower percentage of parents with lower levels of education (SSB, 2020b).

**Table 2:** Parents' education level in the sample.

	***N***	**Percent**	**Valid percent**
Completed grade 10 or less	49	1.1	1.1
Completed grade 13	445	9.6	9.6
Vocational or technical certificate	260	5.6	5.6
Bachelor's degree	1,156	24.9	24.9
Master's degree	2,499	53.8	53.9
Doctoral degree	225	4.8	4.9
Total	4,634	99.8	100.0
Missing	8	0.2	
Total	4,642	100.0	

A total of 4,642 parents across the country answered the survey. When the municipalities of the respondents were divided into groups according to population, they were distributed as shown in Table 3.

**Table 3:** Distribution of respondents in population groups.

	***N***	**Percent**	**Valid percent**
City with more than 300,000 inhabitants^*^	1,030	22.2	22.3
Towns or municipals 65,000–300,000 inhabitants	987	21.3	21.3
Town 15,000–65,000 inhabitants	454	9.8	9.8
Town 5,000–15,000 inhabitants	1,024	22.1	22.1
Village or rural area with less than 5,000 inhabitants	1,129	24.3	24.4
Total	4,624	99.6	100
Missing	18	0.4	
Total	4,642	100	

* *Only Oslo with 700,000 inhabitants*.

A vast majority of Norwegian students go to public schools. In our sample, 4% of the parents reported that their child went to a private school, which is exactly the same percentage as for the country as a whole (SSB, 2020c), again suggesting that the sample reflects the national variation on important measures.

### PA During Homeschooling

The percentages presented in the tables and figures are based on the number of respondents who answered the questions. For the closed-ended questions presented in this article, the missing percentages were very low; specifically, some items were missing regarding parents' education level (0.8%), students' gender (1%), and time spent on PA (0.9%). The percentage of missing answers to the eight items that reflected students' engagement and effort in schoolwork ranged from 0 to 8%. For the two open-ended questions, missing percentages were 35.8% for positive experiences and 32.6% for challenging experiences.

**Figure 1:**
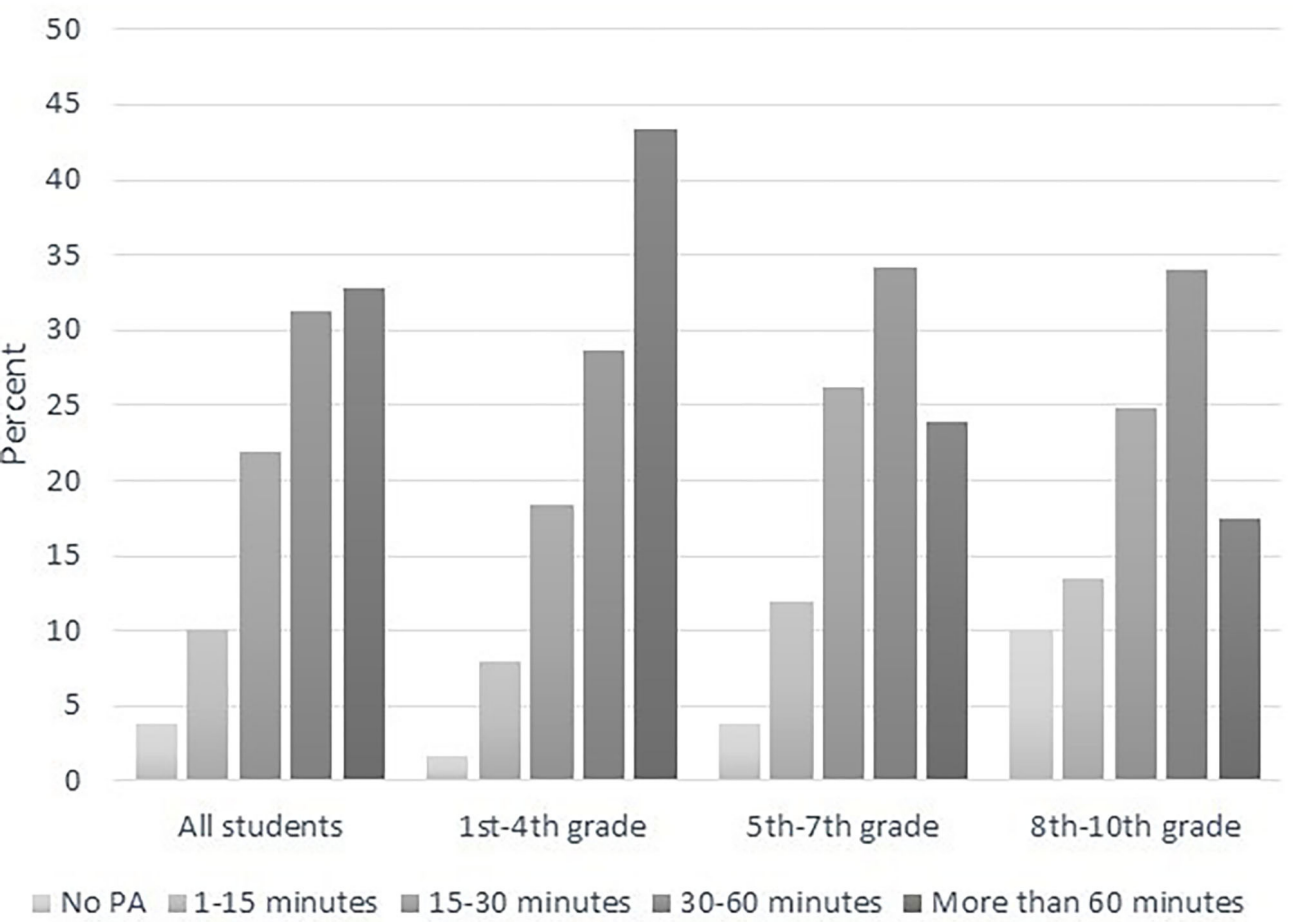
Time spent on daily physical activity among all students and students in three different grade groups.

Figure 1 shows the percentage distribution of responses for each alternative for all parents and for the three grade groups to the question: “How much time does the student spend being physically active during a typical homeschool day, in addition to any PE tasks or exercises given by the teacher as part of homeschool?” The figure shows that only one-third of all parents reported that their child had been physically active for at least 60 min, and more than one-third answered that their child had been active for <30 min. For the group of grades 1–4, however, 43 % of the parents reported that the child had been active for 1 h or more, while this applied to only 17% of the lower secondary students. We ran a χ^2^ test and found that the difference in PA between the three grade groups was statistically significant, χ^2^ (8, *N* = 4.599) = 353, *p* < 0.01.

When we divided the respondents into groups according to the size of the municipality, a χ^2^ test showed that differences between groups based on the size of the municipality was statistically significant, χ^2^ (16, *N* = 4,581) = 392, *p* < 0.01. Further investigation showed differences of less than two percentage points between the groups, in terms of time spent on PA, with one exception: in the big city, 38% reported that their children were physically active for more than 60 min, compared to 30–32% in the other groups.

**Figure 2:**
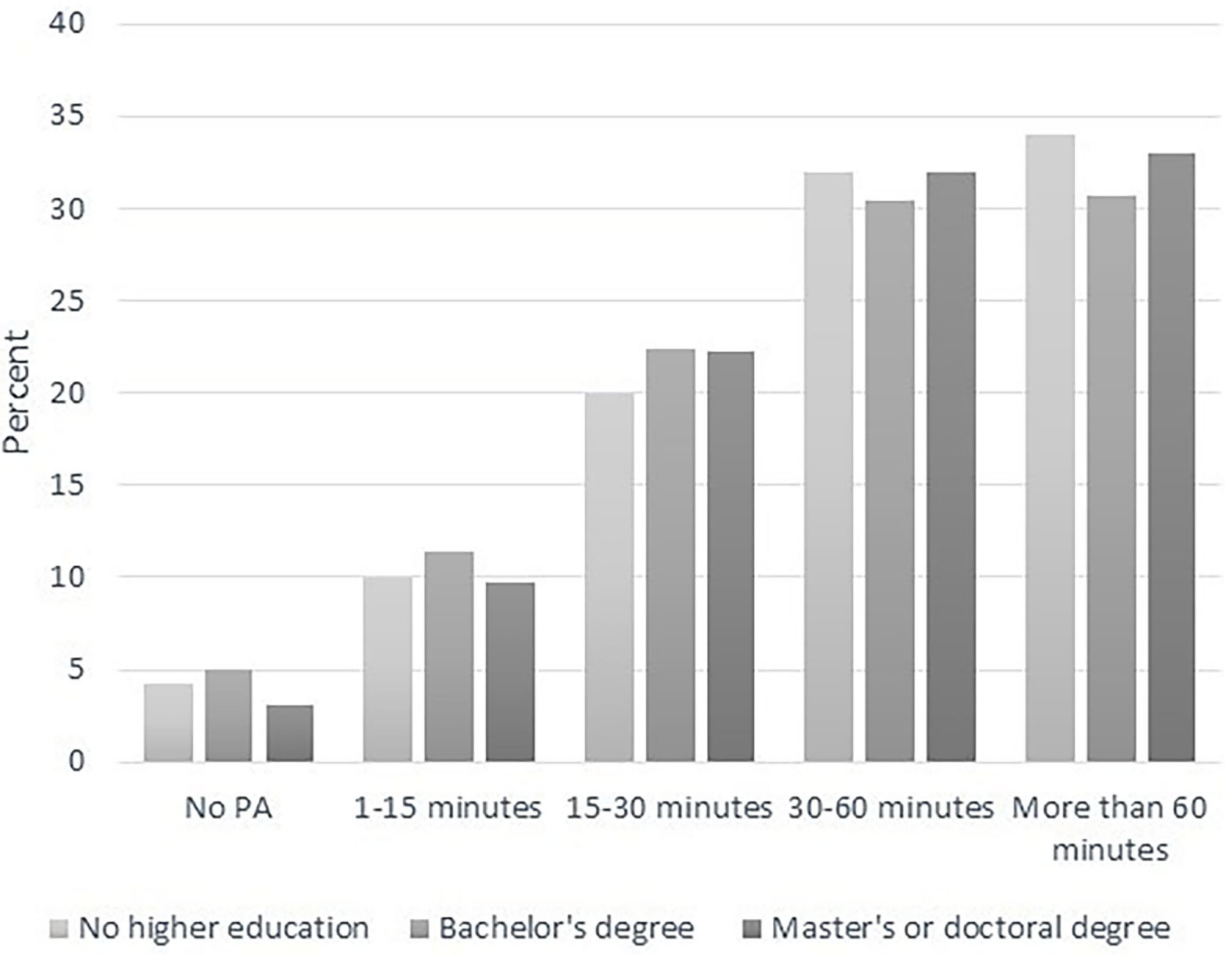
Students' time spent on daily PA reported by three groups of parents according to their educational level.

A χ^2^ test showed no statistical difference in time spent on PA between private and public schools, χ^2^ (4, *N* = 4,596) = 2, *p* = 0.724. Due to the low number of respondents with only 10 years of schooling, we divided the parents into the following three groups to investigate possible differences in students' PA related to the parents' educational level: no higher education, bachelor's degree, and master's or doctoral degree (Figure 2). A χ^2^ test showed no statistical difference between groups of students based on their parents' education, χ^2^ (8, *N* = 4,591) = 13, *p* = 0.13.

**Figure 3:**
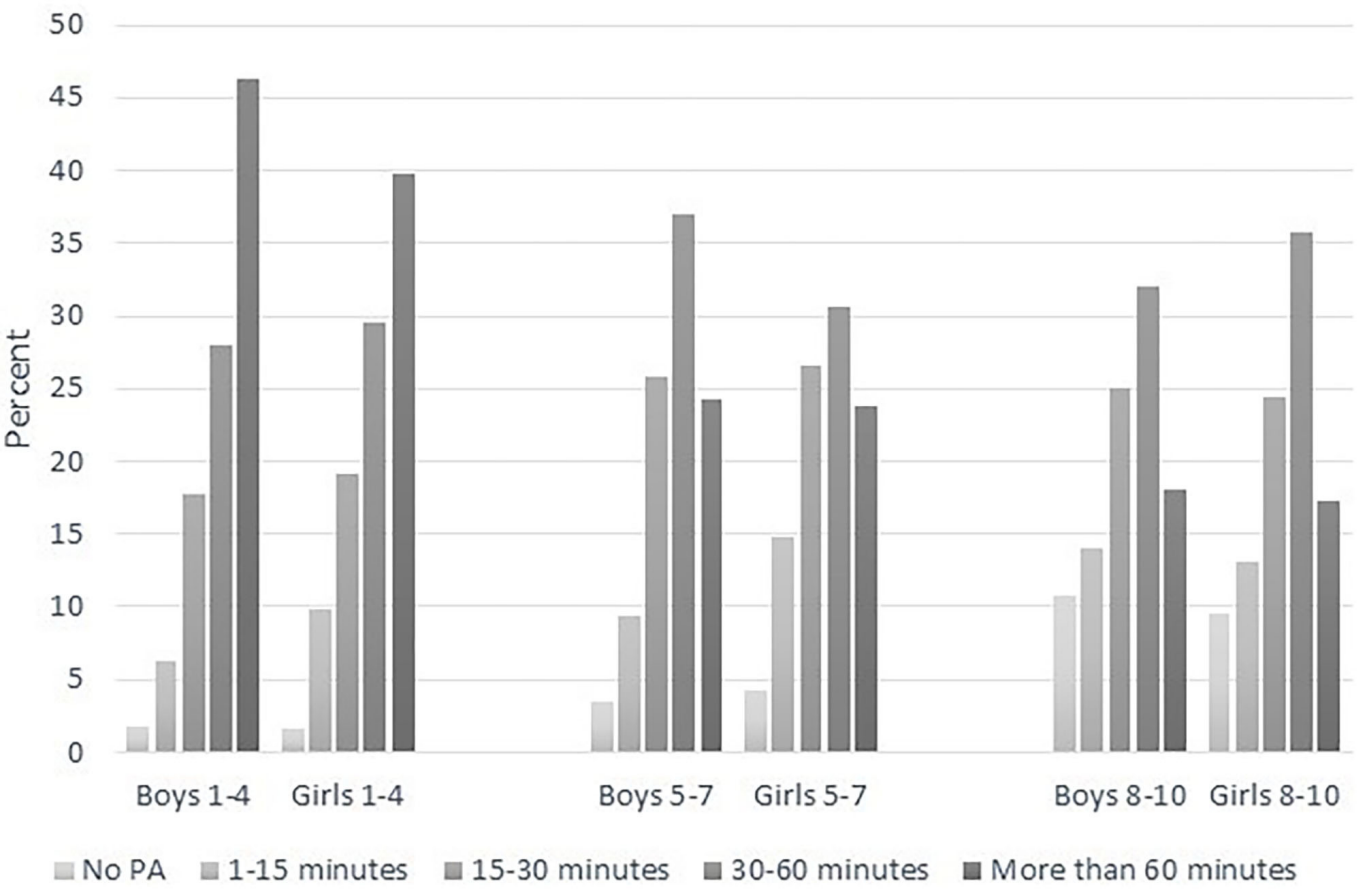
Time spent on daily PA for boys and girls in three grade groups.

Figure 3 shows some instances of gender differences showing that the boys were more active; however, for most response categories, the differences were four percentage points or less. Exceptions were Grades 1–4 where a higher percentage of boys (46%) than girls (40%) were physically active for more than 60 min daily and Grades 5–7 where a higher percentage of boys (61%) than girls (54%) were physically active for at least 30 min per day. In Grades 8–10, however, we found no noteworthy gender differences. As in Figures 1, 3 also emphasizes the gradual decline in time spent being physically active from primary to lower secondary school.

The composite variable which was based on eight items describing students' engagement and effort toward schoolwork was standardized to a total mean of 0 with a standard deviation of 1. Table 4 shows that students in grades 5–7 on average represented the total mean, while students in grades 1–4 on average were scored 0.14 standard deviation below the total mean and students in grades 8–10 were scored 0.33 standard deviation above the total mean. A χ^2^ test showed that the difference in students' engagement and effort in schoolwork between the three grade groups was statistically significant, χ^2^ (64, *N* = 3,596) = 181, *p* < 0.01. Thus, the older the students were, the more engagement and effort they showed in schoolwork, according to their parents. It is imperative to bear these differences between the grade groups in mind when we present the relationship between PA and this variable in the following:

**Table 4:** Mean values for three groups of for parents' reporting of the extent to which students have engagement and effort in schoolwork.

	**Mean**	**Std. deviation**	**Std. error of mean**
1st−4th grade	−0.14	0.98	0.02
5th−7th grade	0.04	0.99	0.03
8th−10th grade	0.33	1.01	0.04

*The total mean was set to 0*.

We ran ANOVAs to test whether students' engagement and effort varied across levels of PA. For students in all the three grade groups the relationship was statistically significant:

1st−4th grade: *F* (4, 1,834) = 9.43, *p* < 0.01.
5th−7th grade: *F* (4, 1,104) = 16.87, *p* < 0.01.
8th−10th grade: *F* (4, 616) = 15.41, *p* < 0.01.

**Figure 4:**
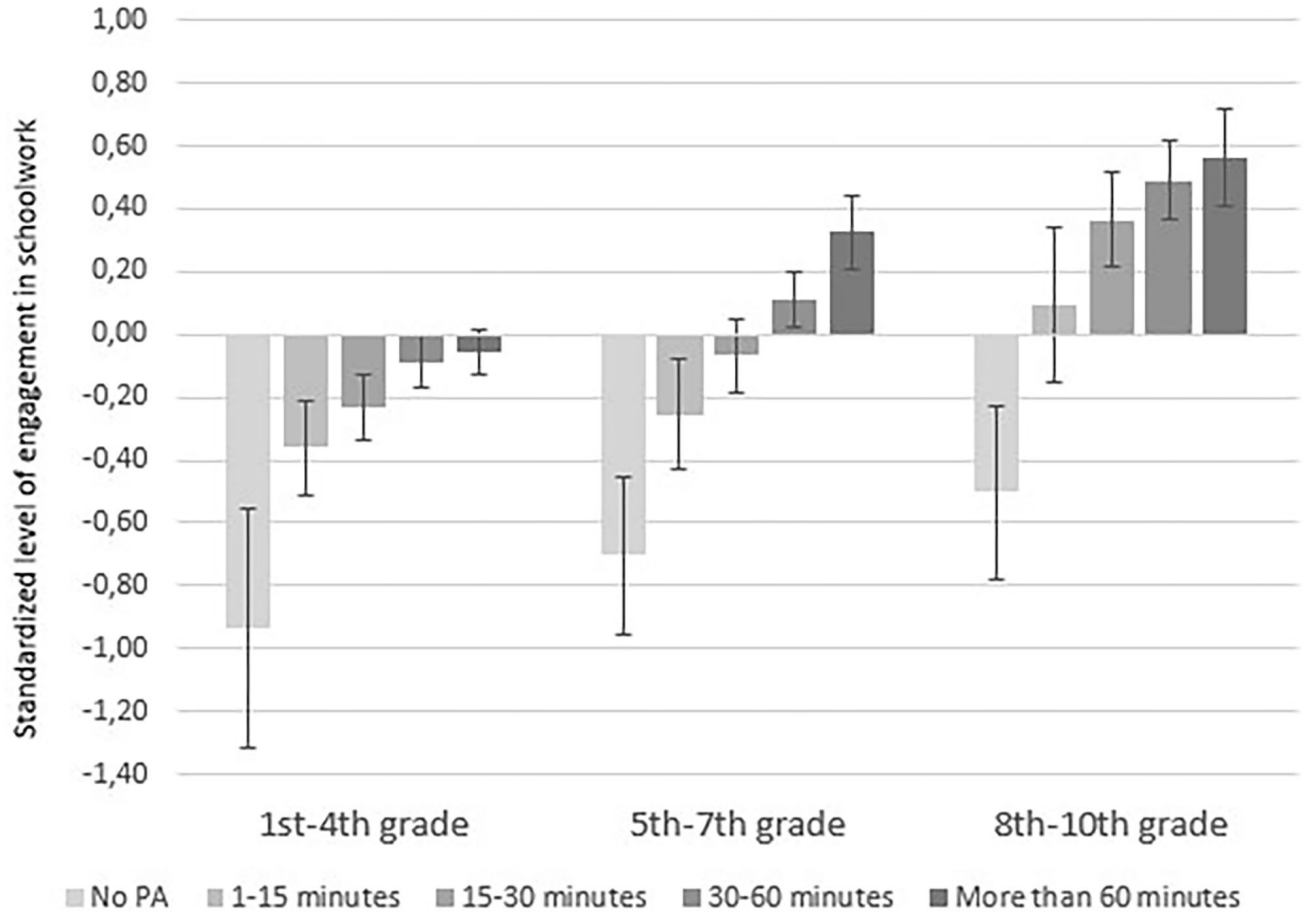
Mean values for three groups of for parents' reporting of the extent to which students show engagement and effort in schoolwork for each of five response categories describing time spent on PA. The total mean was set to 0 and a standard deviation of 1. The 95% confidence interval is marked with 1.96 × SE.

Figure 4 shows the mean values for the concept of engagement and effort in schoolwork related to time spent on PA for the three groups of students. For all grade groups, we found a positive relationship between time spent on PA and students' engagement and effort related to schoolwork. Specifically, the more time they spent being physically active, the more engagement and effort they showed toward schoolwork. Further, students in each grade group who had lower reported levels of PA also were scored lower on engagement and effort in schoolwork than students who were physically active. For students in Grades 8–10, who on average were scored above the total mean in terms of engagement and effort, we found that those who were not physically active were scored significantly below the total mean. This is particularly interesting—and alarming—for this group of students, where one in 10 students were reported to be physically inactive (see Figure 1).

## Short Summary of the Quantitative Analyses

An important finding is thus that, according to the parents, 23% of the students in lower secondary school have been physically active for less than 15 min each day during school hours, with the exception of potential physical exercises given by their PE teacher. Students in Grades 1–4 (5–10 years of age) were the most active, and 43% of these parents stated that their child was physically active for more than 60 min each day. The same was true for only 18% of the students in lower secondary school. Although the youngest students were less engaged in schoolwork than the older children, the analyses showed a clear positive relation between time students spent being physically active during a school day and their engagement and effort toward schoolwork.

## Qualitative Analyses of the Open-Ended Questions

There were two open-ended questions in the survey that allowed parents to elaborate on what they thought had been the main benefits and challenges during the homeschool period. A total of 2,892 (62%) reported different benefits, and 3,032 (65%) reported different challenges. In addition, 174 parents chose to write about PA as either a main benefit or a main problem during the COVID-19 homeschool period. Of these responses, 65 participants reported that homeschooling had been especially positive because their child had more time to be active compared with the time for PA and PE during normal school days. One of the benefits these parents reported had to do with flexibility regarding when different schoolwork should be done, which allowed them to choose the student's pace and approach to schoolwork. Many of the parents saw this flexibility as an opportunity to provide more PA, and they reported that students had more time to play and to be active outdoors, and emphasized that they had more time to go on different walking, hiking, and trekking tours together as a family and to use the natural environment for teaching purposes. The following responses from parents illustrate these ideas:

*We have been able to adapt the homeschooling activity according to both the child's mood and the weather. We have often done the schoolwork down at the shoreline or on a hiking tour in the woods*. (Parent of a boy, Grade 2)
*It was nice that there was some flexibility in relation to when the tasks had to be completed, so we had the opportunity to take a break in the middle of the day and go for longer cross-country skiing trips*. (Parent of a girl, Grade 2)
*He has become more interested in physical activity, including running, strength training, and skiing with us*. (Parent of a boy, Grade 8)
*The student is very interested in the tasks and spends a lot of time on schoolwork, in addition to being able to exercise a lot every day (especially skiing)*. (Parent of a girl, Grade 10).

Quite a few of the parents who reported benefits related to PA highlighted that their child was given the opportunity to do practical work like carpentry, chopping wood, cooking, looking after younger siblings, gardening, and helping with different types of farm work during the period with homeschooling, as shown by the following examples:

[We had] more time together to do practical tasks: cooking, tidying and gardening, sowing seeds, field trips, canoe trips, cabin building, etc. (*Parent of a girl, Grade 2*)
*More time to do physical activity, more time for creative projects, more crafts and practical work (carpenter chopping wood, draining birch sap, putting up a fence, etc.)*. (Parent of a boy, Grade 2)
*She has been helping us more with cooking, and we have been able to sleep outside in the woods on the weekends because we have no other plans*. (Parent of a girl, Grade 4)
*[We had] more time for outdoor activities. It was good to start the day at 09:00, have a good breakfast and a morning walk with the dog every day. (*Parent of a boy, Grade 5)
*My child is a lot outside, walks in the mountains, rides her bike, goes running, jumps on the trampoline, cooks, looks after her little brother, and takes responsibility. And she is social on video meetings too!* (Parent of a girl, Grade 10).

Overall, these findings show that parents who reported positively about PA viewed the opportunity for more flexibility, time for PA, and more family time as main benefits of the homeschooling situation regarding students' PA.

Altogether, 109 of the 174 parents who wrote about PA in the open-ended questions mentioned the lack of PA and increased passivity as main challenges with homeschooling. These parents reported that the period of homeschooling had led to more passivity and a more sedentary lifestyle for their child. Thirty-four of these parents emphasized that their child was playing video games or using a device (e.g., smartphone, computer, and tablet) during most of the day. The responses below illustrate parents' concerns that children were less physically active as a result of lockdown:

*Lots of screens and games, and less physical activity than usual*. (Parent of a boy, Grade 4)
*I wish there were more assignments that did not require students to sit in front of a screen, but be outside. The assignments and the entire school day are now over before 11 am, and the rest of the day is spent on games and TV*. (Parent of a girl, Grade 5)
*There have been no assignments in physical education before this week, and physical activity is really important during these pandemic times. Even though I expect him [the student] to be a little outside in the fresh air every day, it is really hard to get him and his friends to do it. My impression is that they stay inside, gaming. The parents are really not good at getting their children to be outside*. (Parent of a boy, Grade 9).

The remaining 75 responses in this category claimed that it was difficult to both carry out and complete the physical assignments or that it was challenging and hard to motivate their child to be physically active.

*Several of the tasks have required the parents to play games, walk in the woods, etc., which has been very challenging because we have our own job to take care of*. (Parent of girl, Grade 2)
*It is difficult to get the child to perform tasks he does not want, like tasks in Physical Education (PE)*. (Parent of a boy, Grade 6)
*Little physical activity. It had been helpful to have digital activity sessions led by the teacher*. (Parent of a boy, Grade 6)
*It is a challenge that there is so little physical activity during a day. A 14-year-old will not participate in walks in the forest, and there is a lot of sedentary sitting inside. This goes for all his friends, too. From participating in practice three times a week and matches during the weekends to zero physical activity is not a positive development*. (Parent of a boy, Grade 8).

These examples from the open-ended questions elaborate on the quantitative findings, which suggest that the students have been less physically active during the period of homeschooling than they usually are when they attend school. The older the students are, the more they have had a sedentary lifestyle involving little or no PA. As discussed below, the teacher survey also confirmed that PE has not been a priority to the same extent as other subjects, again suggesting less PA for a majority of the students.

## Teacher Survey

In their survey, the teachers were asked to report if any subject was given lower priority than during normal schooling. Multiple answers were possible. Table 5 shows the percentage of teachers who reported that different subject were given lower priority than usual.

**Table 5:** Percentage of teachers who reported that the subject was given lower priority than usual (*N* = 726).

	**Less than with open schools (%)**
Mathematics	8
Language Arts	9
English	9
Natural Science	17
Food and Health	18
Social Science	21
PE	23
Religion	28
Music	30
Arts and Crafts	31

Teachers' responses to the open-ended questions about challenges they faced during homeschooling and remote teaching included statements about the practical difficulties of remotely teaching, PE, music, and arts and crafts, as demonstrated below:

*It is challenging not to be able to ask students to collaborate as I usually would. I use physical activities in my teaching. Now I can't. Some things are more difficult to show students online than when we are in the same physical space together*. (Teacher, Grade 8)
*It has been challenging with arts and crafts, music, voluntary subjects and PE. These subjects rely on what you do in class and require equipment*. (Teacher, Grade 8)
*In PE, it is difficult to get insight into what they do when it comes to exercise and activities*. (Teacher, Grade 8)
*PE has been challenging. To give individual guidance and get an overview of what the students can actually do at home and how they do it (if they even do it). We have to rely on trust and believe that they do things we tell them*. (Teacher, Grade 10)
*PE has been hard. I try to give them different assignments that can be motivating and try to get them to stay active. I have also conducted PE lessons through Teams. I guess it worked really well for some students, but I also have a suspicion that some students do not really participate in these sessions (but appear as visible in the Teams meeting)*. (Teacher, Grade 10)
*Science and PE have been difficult to teach because these are really practical subjects*. (Teacher, Grade 1)
*It has been hard not to be able to help students while they are working. Some things cannot be done. Physical education and physical activity become dependent on the home and the parents' efforts*. (Teacher, Grade 1).

These responses show that several teachers across grades pointed to difficulties in teaching PE when distant from the students. Further, they stated that it was hard to follow up what students were doing, and even the teachers who tried to do PE through platforms like Teams had challenges. As the final teacher quote suggests, PA during closed schools has relied heavily on the parents' initiative or ability to follow up the intended PE provided by the schools.

## Discussion: What Seems to Impact PA During Homeschool?

A key finding about PA during homeschool is that the most active students were those who also worked well with remote schooling in general. This was especially the case with older students. Parents who reported that their children had difficulties following the activities suggested by the school were the same parents who estimated low PA for their child.

The results also suggest that in general, during this period, students had been less physically active than they would have been during open school. In addition to the lack of the expected 1 hour of daily PA schools should provide, students have stayed at home instead of walking to and from school, going in and out of classrooms, and playing in the schoolyard during breaks. Further, the vast majority of 6–15-year-olds do organized sports one or more days a week (Hammer, 2017; Bakken, 2019), which they have not been able to because of the shutdowns. Due to these factors, it is plausible that prior to the pandemic, students would have attained 1 hour of PA on an average weekday, or would have had the opportunity to do so before the restrictions imposed because of COVID-19.

For the younger children, parents reported higher degrees of PA than the parents of older children, which is consistent with prior research showing that younger students are more active (Hammer, 2017; Bakken, 2019). This finding also reflects that teachers in the lower grades have followed up the students in a less structured way, with few requirements for attendance, which has given families the possibility to prioritize PA every day. An important point to be made here is that there was great variation in what kind of educational activities the teachers provided for their students (Roe et al., 2020). Given that the parents viewed “flexibility to structure the day” as one of the main advantages, especially for younger children, it is reasonable to assume that a lot of the PA among the youngest children was initiated by the parents, not the teachers.

Prior research has determined that PA plays an important role in both physical and psychosocial health and wellbeing for children and young people (Biddle et al., 2019), and there is ample evidence that a sedentary lifestyle in students is associated with chronic diseases later in life and other health-related risk behaviors such has unhealthy dietary patterns (Carson et al., 2016; Tremblay et al., 2016). Research has also shown that the amount of PA declines with age and is associated with socioeconomic background (Hammer, 2017; Bakken, 2019). Our survey suggests that the home situation (e.g., two parents at home, flexibility from the school) allowed some families to really prioritize PA during COVID-19 outbreak. Further, the survey shows that, although the youngest students were less engaged in schoolwork than the older children, there was a clear positive relation between time students spent being physically active during a school day and their engagement and effort toward schoolwork. It seems that, for many families, the time during the COVID-19 pandemic provided more flexible days, where some systematically chose to include more PA. Some parents reported in the open-ended questions that their child was happier and more content than normal during homeschooling.

An important implication of our findings is that, in times of homeschooling, policymakers and educators alike need to discuss whether the degree of PA should be a result of parents' initiative or if the schools to a larger degree could provide students with tasks and assignments requiring them to systematically engage in PA at home. Previous research has shown how socioeconomic status is directly associated with health and PA, making this question all the more important. A major point to be made about the homeschool situation in Norway is that the types of activities schools have provided have varied greatly, as has how much different teachers have followed up their students (Roe et al., 2020). Some parents in the survey reported that their children were asked to use specific apps in PE during homeschool, but results from both the parent and the teacher survey as well as previous research on teachers' digital repertoires (Røkenes and Krumsvik, 2016; Blikstad-Balas and Klette, 2020) have indicated that there is room for improvement when it comes to using all the digital possibilities for educational purposes. Digital tools enable, for example, joint digital workout sessions, digital tracing of exercises, and a number of instructional videos that students could use. Previous research from Norway has suggested that teachers rarely use these forms of digitally innovative ways of teaching (Blikstad-Balas and Klette, 2020). Our materials present a clear trend of teachers prioritizing tasking students with individual assignments that require sedentary work.

This study suggests that many students were given too much responsibility for their own PA during this period, making PA dependent on their parents' priorities and the parents' possibilities to follow up. A pedagogical implication from this work is that teachers should consider providing their students with more digital workout sessions and instructional videos, as well as using apps and tracking devices that actually document students' degree of PA. Given that the digital infrastructure is present, we are somewhat surprised that the digital repertoire of the teacher revolved so heavily on giving individual tasks and so little on joint real-time instruction. A number of digital possibilities exist to measure students' activity, given that all relevant permissions are provided and rules such as the General Data Protection Regulation are followed. Such possibilities could be used to ensure that PA is not something that relies on parent initiative alone. This discussion is particularly important as WHO (2020) predicts more situations where homeschooling could be necessary in the future.

An important limitation with the survey data we presented in this article is that the survey was developed as a holistic measure of the homeschool experience for parents and teachers, not solely focusing on different aspects of PA during homeschool. It also relies on parents' perceptions of how physically active their children have been. Another significant limitation is that we lack accurate measures of how active the students would be during normal school days. Further, we also lack systematic and representative data from PE teachers mapping which digital tools they used in PE and how frequently these were used at different grade levels. Despite these limitations, we believe the present study provides important insight into how parents have prioritized PA in the time of COVID-19 and how the PA levels of children during homeschooling were greatly influenced by their parents' initiative.

## Data Availability Statement

The raw data supporting the conclusions of this article will be made available by the authors, without undue reservation.

## Author Contributions

All authors listed have made a substantial, direct and intellectual contribution to the work, and approved it for publication.

## Conflict of Interest

The authors declare that the research was conducted in the absence of any commercial or financial relationships that could be construed as a potential conflict of interest.
